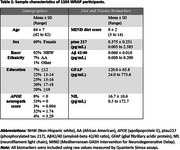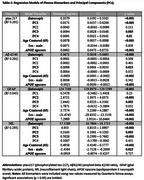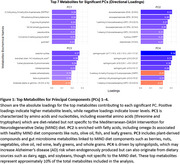# Diet‐Driven Metabolite Patterns Link the MIND Diet to Dementia Biomarkers

**DOI:** 10.1002/alz70860_106699

**Published:** 2025-12-23

**Authors:** Diandra N. Denier‐Fields, Ruocheng Dong, Briana L Rocha, Nathaniel A. Chin, Ramiro Eduardo Rea Reyes, Rachael E. Wilson, Erin M. Jonaitis, Rebecca E. Langhough, Sterling C Johnson, Corinne D. Engelman

**Affiliations:** ^1^ University of Wisconsin‐Madison, Madison, WI, USA

## Abstract

**Background:**

Plasma biomarkers such as phosphorylated tau 217 (ptau217), amyloid‐beta 42/40 ratio (Aβ42/40), glial fibrillary acidic protein (GFAP), and neurofilament light chain (NfL) are valuable markers of Alzheimer's disease (AD), neuroinflammation, and neurodegeneration, respectively. These biomarkers serve as cost‐effective and accessible tools to assess early indicators of dementia risk. The Mediterranean‐DASH Intervention for Neurodegenerative Delay (MIND) diet, a modifiable lifestyle factor, has been associated with reduced dementia incidence. Plasma metabolomics offers an objective alternative to dietary questionnaires by capturing nutrient absorption and physiological impacts.

**Method:**

This study investigated associations between the MIND diet score, plasma metabolites, and plasma biomarkers within 1,104 participants cognitively unimpaired at baseline from the Wisconsin Registry for Alzheimer's Prevention (WRAP) cohort. Plasma metabolites serving as proxies for diet were identified through an initial association analysis of nearly 4,900 observations, including over 1,800 participants from WRAP and the Alzheimer's Disease Research Center. This analysis identified 72 plasma metabolites significantly associated with the MIND diet score. Missing metabolite values were imputed using the mean before Principal Component Analysis (PCA) was performed on these metabolites to derive metabolite patterns, which were subsequently tested for associations with plasma biomarkers measured within six months of MIND diet questionnaire completion and plasma metabolomics sample collection. General linear regression models were used to assess association, adjusting for age, sex and apolipoprotein E (*APOE*).

**Result:**

Sample characteristics are shown in Table 1. The first four principal components (PCs) explained 38% of the variance in the MIND diet‐associated metabolites. Figure 1 presents the top seven metabolites for each PC. Regression models (Table 2) identified significant positive PC‐biomarker associations: PC1 with ptau217 and NfL; PC2 with GFAP and NfL; PC3 with all four biomarkers; and PC4 with ptau217. Mediation analysis will examine whether these PCs mediate the relationship between the MIND diet and plasma biomarkers.

**Conclusion:**

MIND diet‐associated plasma metabolite groups correlate with plasma biomarkers of AD and neurodegeneration. These groups of metabolites include essential amino acids, omega‐3 fatty acids, plant and gut microbe‐derived metabolites, and sphingolipids. These findings highlight the potential importance of dietary interventions in modulating metabolic pathways linked to dementia biomarkers.